# Recent progress of microfluidic chips in immunoassay

**DOI:** 10.3389/fbioe.2022.1112327

**Published:** 2022-12-23

**Authors:** Kaimin Wu, Xuliang He, Jinglei Wang, Ting Pan, Ran He, Feizhi Kong, Zhenmin Cao, Feiye Ju, Zhao Huang, Libo Nie

**Affiliations:** ^1^ Hunan Key Laboratory of Biomedical Nanomaterials and Devices, Hunan University of Technology, Zhuzhou, China; ^2^ Zhuzhou People’s Hospital, Zhuzhou, China

**Keywords:** microfluidic chip, immunoassay, fluorescence, chemiluminescence, surface-enhanced Raman spectroscopy, electrochemistry

## Abstract

Microfluidic chip technology is a technology platform that integrates basic operation units such as processing, separation, reaction and detection into microchannel chip to realize low consumption, fast and efficient analysis of samples. It has the characteristics of small volume need of samples and reagents, fast analysis, low cost, automation, portability, high throughout, and good compatibility with other techniques. In this review, the concept, preparation materials and fabrication technology of microfluidic chip are described. The applications of microfluidic chip in immunoassay, including fluorescent, chemiluminescent, surface-enhanced Raman spectroscopy (SERS), and electrochemical immunoassay are reviewed. Look into the future, the development of microfluidic chips lies in point-of-care testing and high throughput equipment, and there are still some challenges in the design and the integration of microfluidic chips, as well as the analysis of actual sample by microfluidic chips.

## 1 Introduction

Microfluidic technology refers to the process or manipulation of a small amount of fluid (10-9-10-18 L) using microchannels with tens to hundreds of microns (Abe et al., 2010; Alix-Panabières and Pantel, 2014; Huang L. et al., 2021; Bai et al., 2022). It is a new research field developed from the technologies and principles of chemistry, physics, biology, materials science, fluid mechanics and microelectronics (Deng et al., 2013; Sackmann et al., 2014; Hamdallah et al., 2020). In the early 1990s, Manz et al. carried out the research on chip electrophoresis and first proposed the concept of microcomplete analysis system (Manz et al., 1990).

Microfluidic chip, also called microfluidic chip lab or lab-on-chip (LOC), is a technology platform that integrates the sample processing, reaction, separation, detection and other routine operations involved in chemical and biological experiments on a chip of several square centimeters to achieve low consumption, rapid and efficient analysis. Microfluidic chip is characterized by the its microstructure to accommodate the fluid, such as micro channels, micro reaction chambers and other micro functional components, resulting in a series of special effects of micro fluid, such as laminar flow effect, surface tension and capillary effect, rapid heat conduction effect and diffusion effect, which is conducive to accurate fluid control and rapid reaction. Due to the micron-scale structure, the fluid displays special properties that are different from those in the macroscopic scale, which leads to unique analytical performance (Li et al., 2013b; Mou et al., 2016; Liu H. et al., 2017; Zhang Z. et al., 2022). Compared with traditional analysis methods, the prominent advantage of microfluidic chip lies in the flexible combination and integration of various unit, realizing the automatic operation, high throughput detection and low reagent consumption, preventing human interference and pollution, as well as completing efficient repeated experiments. Microfluidic chip is also of good compatibility that easy to integrate with other technical devices (Daw and Finkelstein, 2006). Using microfluidic chip technology, hundreds of samples can be simultaneously analyzed in a few minutes or even less time, and the whole process of sample pretreatment and analysis can be realized automatically (Li et al., 2013a; Liu et al., 2013; Lu et al., 2021).

With the development of materials science, the materials of microfluidic chips have emerged in a wide range from silicon, glass to paper-based, hydrogels, and polymers. At the same time, the preparation technology of microfluidic chip is also booming, including screen printing, inkjet printing, 3D printing and so on. Some advanced processing technologies, such as femtosecond laser processing technology and two-photon 3D printing technology, also provide more possibilities for the production of high-precision microfluidic chips.

Microfluidic chip technology has brought a subversive breakthrough in biomedical field. Due to the advantages of small reagent consumption, short reaction process, high sensitivity and low cost, microfluidic chips are easy to be developed into portable instruments and have significant advantages of real-time on-site detection, which can be widely used in gene, protein and microbiology assay. Microfluidic chip is also potentially used in portable instruments and point-of-care testing (POCT), which can be widely used in the field of biomedicine (Hale et al., 2010; van de Sandt et al., 2012; Wang H. et al., 2021; Huang Q. et al., 2021; Goldstein et al., 2021; Jalili et al., 2021; Ma et al., 2021; Yin et al., 2021; Dornhof et al., 2022; Sun et al., 2022; Yang et al., 2022).

## 2 Preparation of microfluidic chip

### 2.1 Materials of microfluidic chip

In the preparation of microfluidic chips, the durability, manufacturability, transparency, biocompatibility, as well as the possibility of surface functionalization of the materials must be considered (Pan et al., 2017; Sticker et al., 2020; Niculescu et al., 2021). At present, the materials of microfluidic chips include silicon, quartz, glass, polymer, hydrogel, paper and so on (Qin et al., 1998).

Silicon is one of the favored materials for microfluidic chip preparation, which is easy to obtain and has good chemical compatibility and thermal stability (Singh et al., 2011; Martins et al., 2018). At the same time, silicon has been the preferred material for microfluidic platform preparation for decades due to its simplicity of manufacturing, easy to be surface modified and the semiconductor properties (Nielsen et al., 2020).

Glass and quartz are also widely used as the materials of microfluidic chips due to their low cost and good biocompatibility, with the surface modification based on silicon hydroxyl (Jiang X. et al., 2020). In addition, glass is of excellent light transmittance which is convenient for optical detection (Hwang et al., 2019).

Polymer is a kind of important material used in microfluidic chip because of its variety, low cost, simple manufacturing process, good transparency, and modifiable performance. Polymer used in microfluidic chip involves of thermoplastic polymer, thermosetting polymer and solvent volatile polymer. Thermoplastic polymer includes polymethyl methacrylate (PMMA), polycarbonate (PC) and polyethylene (PE), etc. Thermosetting polymer includes polydimethylsiloxane (PDMS), epoxy resin and polyurethane (PU), *etc.* Solvent volatile polymer includes acrylic, rubber and fluorine plastics, *etc.* PDMS is hydrophobic organosilicon material, which has the advantages of no byproduct, low stress and modulus, excellent physiological inertia, and remaining elastic within the temperature range of −40–210°C (Syed et al., 2014). PMMA is also known as plexiglass, with the properties of easy processing, strong corrosion resistance, good insulation and high transparency. The density of PMMA is much less than that of glass, but the strength of PMMA is 10 times stronger than that of glass (Gabriel et al., 2014).

Hydrogel has become one of the matrix materials of microfluidic chips (Liu M. et al., 2017; Yang F. et al., 2018; Jing et al., 2019; Mofazzal Jahromi et al., 2019; Nielsen et al., 2020), which usually have a hydrophilic porous structure, and its porous structure is filled with water molecules, allowing biomacromolecules to pass through (Lee et al., 2014; Chen et al., 2016; Liu M. et al., 2017; Chen et al., 2017; Xu et al., 2019; Huang et al., 2020; Vera et al., 2021).

Paper materials have the history of use more than two thousand years, which usually has a loose porous hydrophilic structure. Water-based liquid dropped on the surface of the paper can flow autonomously in the paper fiber by capillary forces. The application of paper materials in microfluidic chips is mainly inspired by the application of paper chromatography in traditional chemical detection methods (Martinez et al., 2007; Hellmann, 2008). The microfluidic analysis platform prepared from paper (such as chromatography paper and nitrocellulose film) is called paper-based microfluidic chip, also known as paper chip. Microfluidic paper-based chip is a miniature laboratory analysis system that uses paper substrates to replace the traditional substrates of microfluidic systems. By establishing hydrophilic/hydrophobic channels and analysis units on the surface, the sample on the chip carries out self-driven flow and corresponding reactions to realize sample pretreatment, separation, purification, and detection (Wu et al., 2016; Wu et al., 2017). Paper-based microfluidic chips are characterized by low cost, easy processing, disposable use and no external fluid pump, which greatly simplifies the requirements of chip structure for auxiliary equipment (Lin et al., 2020; Liu et al., 2020). In recent years, paper-based microfluidic chip has been applied to a certain extent in life science and medicine, food safety, environmental monitoring and other fields (Qi et al., 2018; Jiang H. et al., 2020; Yang et al., 2020). Except the advantages, there are still many constraints in the application of paper-based microfluidic chips. For example, the sample is volatile in the channel or in the flow process, resulting in the loss of the sample. The precision of the paper chip, as well as the sensitivity of colorimetric detection is relatively low. In addition, the sample may leak in the process of circulation. The performance of paper chip can be improved by selecting the appropriate paper substrate, reasonably designing the fluid channel and employing optimal processing method.

### 2.2 Fabrication of microfluidic chips

Different fabrication methods of microfluidic chips are adopted according to properties of the substrates. The surface of silicon materials can be modified by silanization to reduce “non-specific” adsorption (Wu et al., 2009; Muthusubramaniam et al., 2011). However, silicon materials also have the shortcomings such as poor light transmittance, fragility and poor electrical insulation. The process methods of silicon chip are mainly photolithography and etching (Chow et al., 2004). Due to their special surface properties, glass and quartz are difficult to be treated directly. The common processes are photoetching and lithography, which are divided into gluing, exposure, development, etching, degluing, drilling, and sealing (Nakanishi et al., 2001; Qiu et al., 2020). The application of glass and quartz chips in microfluidic devices is limited due to the high cost of micromachining, time consumption, and the requirement of a clean room in preparation process (Tang et al., 2021). The fabrication of polymer microfluidic chip mainly adopts hot pressing, moulding method, injection moulding method and so on. Only after the preparation of microchannel can the sample tube be modified, installed and sealed (Chen et al., 2001; Krishnan et al., 2004; Wu et al., 2005; Liu et al., 2009). The preparation of hydrogel microfluidic chip is significantly different from that of silicon/glass and polymer materials, which is mainly prepared by UV laser or 3D printing (Decock et al., 2018).

The preparation of paper-based microfluidic chips involves various methods to construct a hydrophilic/hydrophobic channel on the paper-based surface so that the dripping sample can flow directly through the preset channel. Initially, ultraviolet lithography was used to treat the paper and construct hydrophobic channels on the surface of cellulose paper (Martinez et al., 2007). With the development of technology, the processing methods of paper-based microfluidic chips have been greatly improved currently, which include inkjet printing, inkjet etching, wax printing, wax immersion, plasma treatment, etc., (Carrilho et al., 2009; Abe et al., 2010; Li et al., 2010a; Li et al., 2010b; Chitnis et al., 2011).

## 3 Application of microfluidic chip in immunoassay

Microfluidic chip not only realizes low reagent consumption and high throughput detection, but also has obvious advantages of easy integration and good compatibility with other technical equipment. Nowadays, microfluidic chips are widely used in bioassay. Immunoassay detects the analytes (mainly proteins) based on antibody-antigen reaction. Common immunoassay methods include enzyme linked immunosorbent assay (ELISA), radioimmunoassay, fluorescence immunoassay, chemiluminescence, etc. (Lai et al., 2018a; Lai et al., 2018b). Traditional immunoassay relies on large equipment and skilled operators, with complicated operation, expensive reagents, long assay time and low sensitivity. At the same time, with the demand increasing for real-time detection, clinical diagnosis need a high sensitivity, high accuracy, rapid, portable and real-time immunoassay method. The combination of immunoassay and microfluidic technology can greatly overcome the shortcomings of traditional immunoassay. For example, the high surface-to-volume ratio of micro-channels is able to accelerate the antibody-antigen binding to shorten the reaction time. Microfluidic immunoassay exhibits a lot of advantages such as fast response, low reagent consumption, high-throughput and portability (Zheng et al., 2020; Wang Y. et al., 2021; Reis et al., 2021; Rodriguez-Moncayo et al., 2021). Researchers have developed a variety of microfluidic immunoassay chips by combining microfluidic technology with various detection techniques such as fluorescent, chemiluminescence, surface-enhanced Raman spectroscopy (SERS), and electrochemical methods and so on.

### 3.1 Fluorescent microfluidic immunoassay

**FIGURE 1:**
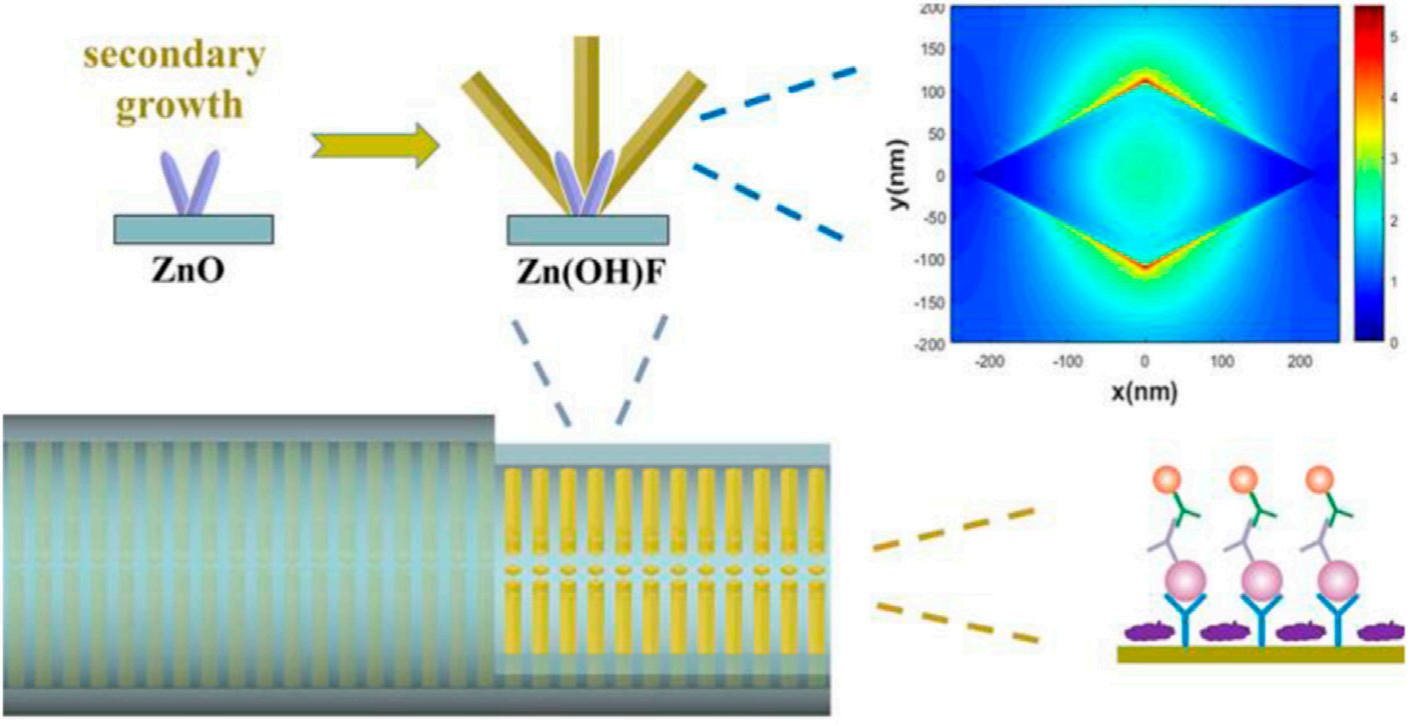
Zn(OH)F array-based microfluidic fluorescence assay (Zhao et al., 2021).

Among all kinds of detection methods, immunofluorescence detection is a technology with high sensitivity and easy to realize real-time diagnosis. In fluorescence immunoassays, fluorescent materials such as fluorescent dyes, quantum dots (QDs), upconversion nanoparticles (UCNPs), fluorescent microspheres, aggregationinduced emission (AIE) luminogens are labeled with antigens or antibodies to achieve quantification of analytes, which can be used in the combination of fluorescence immunoassay with microfluidic chips (Zhou L. et al., 2016; Mao et al., 2016; Zhou et al., 2017; Li et al., 2019; Tan et al., 2019; Xie et al., 2019; Ding et al., 2020; Zuo et al., 2020; Chen Y. et al., 2021; Gong et al., 2021). To improve the sensitivity of fluorescent microfluidic immunoassay, integration of nanostructures with microchannels is a promising approach. At present, ZnO has been broadly used in fluorescence immunoassays because of its fluorescence amplification ability towards biomolecular (Hu et al., 2015; Liu et al., 2016; Piguillem et al., 2020). Guo et al. grew ZnO nanowires in microfluidic channels for enhanced fluorescent detection of cancer biomarkers. Due to the increase of the binding surface area and the fluorescence amplification of ZnO nanowires, the nanostructure integrated microfluidic chip attained a LOD of 1 pg/ml and 100 fg/ml in human α-fetoprotein (AFP) and carcinoembryonic antigen (CEA) detection, respectively (Guo et al., 2018). Duan’s group designed a micro/nanostructure integrated microfluidic chip by combining ZnO nanorod arrays with *in situ* zeolitic imidazolate framework-8 (ZIF-8) coating for synergetic enhanced fluorescent detection. Taking advantage of the synergetic fluorescence enhancement of ZnO nanorod and ZIF-8 towards organic fluorescence labels, the microfluidic fluorescence detection of CEA exhibited a LOD as low as 0.01 pg ml^−1^ and a linear range of 0.01–100 pg ml^−1^ (Zhao et al., 2020). Furthermore, they proposed a rhombus-like Zn(OH)F array-based microfluidic device for fluorescence detection of human epididymis-specific protein 4 (HE4). The results showed that the fluorescence enhancement factor of the Zn(OH)F arrays toward Cy3 is approximately 4-fold that of the ZnO nanorod arrays, with a LOD of 9.3 fM and a linear range of 10 fM to 100 pM (Figure 1) (Zhao et al., 2021).

**FIGURE 2:**
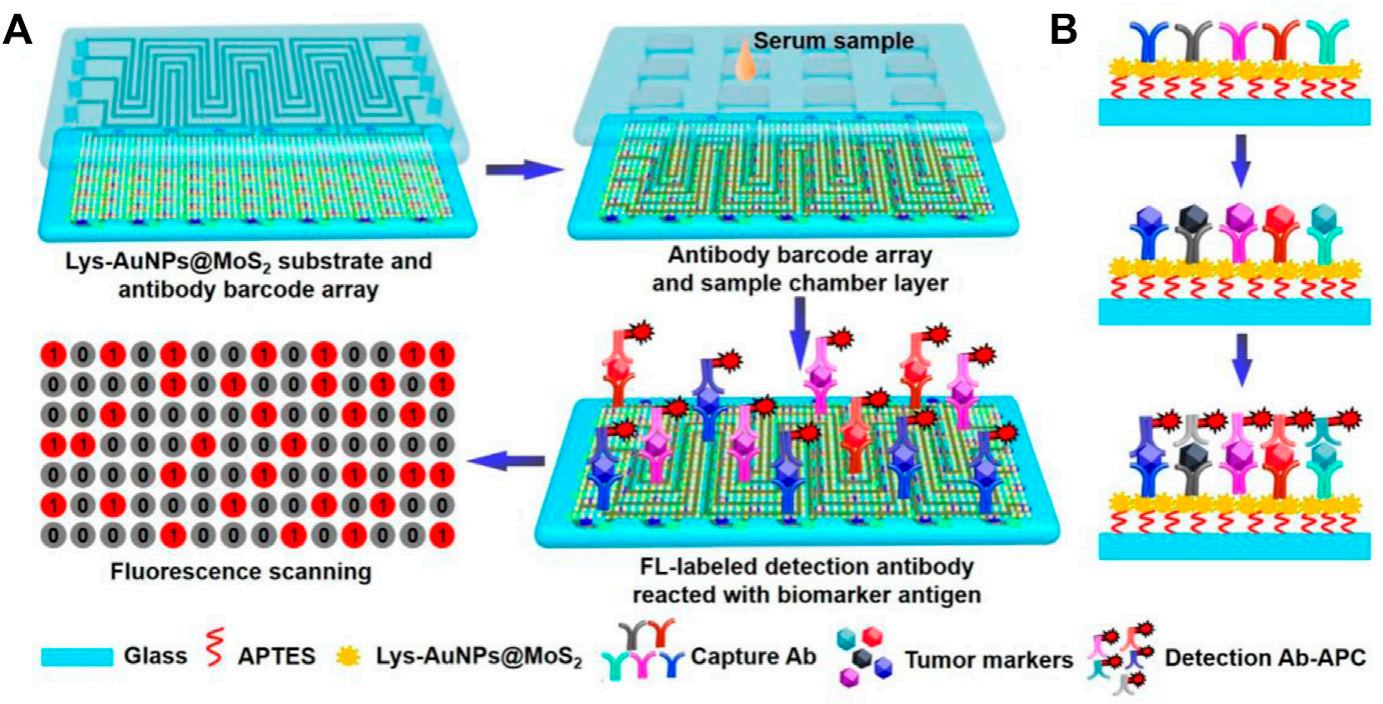
Schematic diagram of immunoassay microfluidic chip based on Lys-AuNPs@MoS_2_ substrate. **(A)** Self-assembly of the Lys-AuNPs@MoS_2_ substrate. **(B)** Mechanism of microfluidic incubation.

High throughput is an important developing direct for bioassay. To achieve high throughput immunoassay, Duan et al. combined fluorescence detection with microfluidic chips for rapid semi-quantitative detection of serum biomarker mesothelin, which allowed the simultaneous measurement of four samples with three repeats (Duan et al., 2019). Qiu et al. developed a fluorescent microfluidic immunoassay biochip based on self-assembly of Lys-AuNPs@MoS_2_ nanocomposites with large contact surface, excellent stabilities and multiple binding sites (Figure 2). In this system, 60 samples such as inflammatory factors and cardiovascular biomarkers were simultaneously detected within 40 min with a limit of detection (LOD) of several to tens pg/mL (Qiu et al., 2022).

**FIGURE 3:**
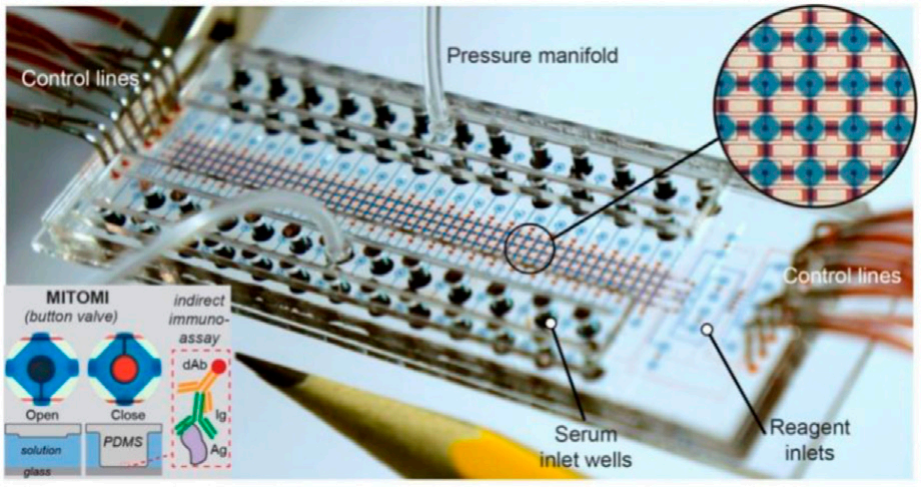
Microfluidic device using MITOMI for COVID-19 antibody detection (Rodriguez-Moncayo et al., 2021).

Roberto et al. developed a microfluidic device with a button valve located on each microchamber (Figure 3). Fluorescence signals were measured for four types of severe acute respiratory syndrome coronavirus 2 (SARS-COV-2) immunoglobulin in parallel from 50 serum samples, achieving a sensitivity of 95% and a specificity of 91%. In the third week after the onset of symptoms of COVID-19 infection, the sensitivity and specificity can reach 100% when assessing the serums of patients (Rodriguez-Moncayo et al., 2021).

Although with high sensitivity, the fluorescence signals of fluorescent microfluidic immunoassay are usually collected by fluorescence microscope or scanner. The application of these large instruments will increase the experimental cost and affect the portability of microfluidic devices. Therefore, the miniaturization of fluorescent microfluidic device is a goal to realize the whole process of sample pretreatment, immunobinding reaction and fluorescence signal collection on the chip, so as to realize the portable microfluidic immunofluorescence analysis system.

### 3.2 Chemiluminescent microfluidic immunoassay

**FIGURE 4:**
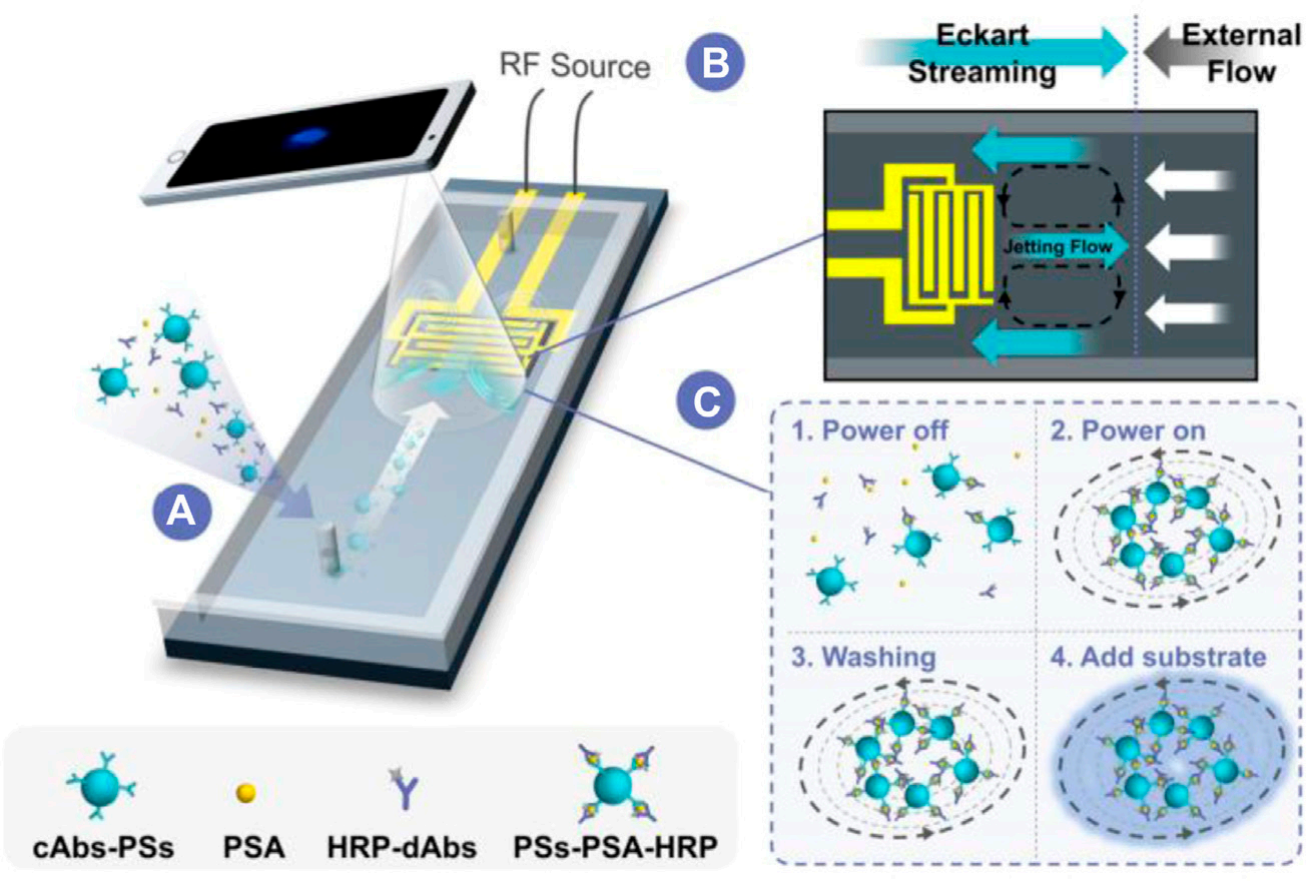
Acoustically enhanced microfluidic smartphone detection platform. **(A)** The platform enhances biomolecular binding and enriches the formed immunocomplexes simultaneously. **(B)** The opposite Eckart streaming and external flow determine the size of the vortex and the compactness of the particles in the vortex. **(C)** The interactions between reagents were enhanced by streaming and particle trapping, washing, reaction, and sensing are achieved on the same chip at continuous flow.

The chemiluminescence immunoassay (CLIA) which combine chemiluminescence detection with a specific immunosorbent assay is widely used in clinical diagnostic (Zhou B. et al., 2016; Yao et al., 2016). The conventional CLIA is limited by the requirement of large equipment, cumbersome operation process, and high consumption. Integration of CLIA with microfluidic chip is promising to offer a simple, low reagent consumption and inexpensive platform for immunoassay, and promote high throughput screening and POCT diagnostics. Huang et al. developed an active droplet-array (ADA) microfluidics-based CLIA system for procalcitonin detection, which consists of a compact microchip analyzer and microfluidic chips with preloaded reagents. The detection can be completed automatically in 12 min, with a LOD of 0.044 ng ml^−1^ and a detection range from 0.044 to 100 ng ml^−1^ (Huang et al., 2022). Smartphone-enabled microfluidic CLIA is a promising portable system for POC applications, but it is suffered from the rather weak chemiluminescent light emitted from a limited sample volume in the microchannel when using the smartphone as a detector. Chen et al. proposed a novel acoustic streaming tweezers-enabled microfluidic CLIA (Figure 4). In this design, probe particles were enriched and the biomarker capture capability was enhanced under high-speed microscale vortexes, which increased the local chemiluminescent intensity and enable the direct capture of light signal by a common smartphone camera. The system reached a LOD of 0.2 ng/ml and a large linear range from 0.3 to 10 ng/ml for prostate-specific antigen (PSA) detection (Chen X. et al., 2021).

**FIGURE 5:**
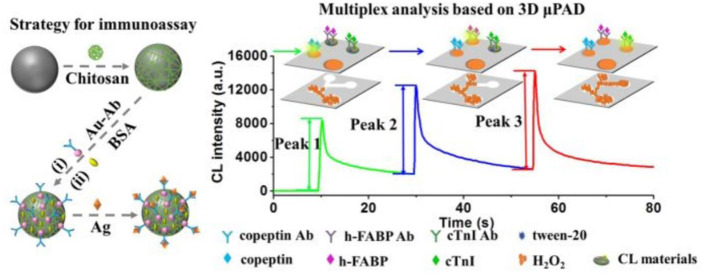
Schematic illustration of multiplex CLIA based on Co^2+^-ABEI-Fe_3_O_4_@void@C and 3D µPAD (Yang et al., 2019).

Up to now, multiplex CLIA for sensitive and simultaneous detection of biomarkers still remains a great challenge. Two strategies of CL multiplex microfluidic immunoassay in one detection run have been reported, including temporal-resolved and spatial-resolved detection. For temporal-resolved CLIA strategy, solution migration delays are created in microfluidic paper-based analytical devices (μPADs) to generate temporal-resolved CL signals for multiplex analysis. For spatial-resolved CLIA strategy, spatial-resolved CL arrays were fabricated by spotting different antibodies at defined positions for simultaneous determination, and the CL imaging signals are recorded simultaneously by a cooled low-light charge-coupled imaging device (CCD). Cui’s group developed a CLIA strategy for simultaneous detection of copeptin, heart-type fatty acid binding protein (h-FABP), and cardiac troponin I (cTnI) by using Co^2+^/N-(aminobutyl)-N-(ethylisoluminol) (ABEI) functionalized magnetic carbon composite (Co^2+^-ABEI-Fe_3_O_4_@void@C) as an interface and a three-dimensional paper-based analytical devices as a detection system (Figure 5). The antigen (Ag) were captured by the antibody on the sensing interface to increase CL intensity due to the catalysis of −COO–existing in Ag. After injecting H_2_O_2_, three time-resolved CL signals were generated in one CL detection run by virtue of time-delayed transport of H_2_O_2_ to different detection zones. The detection limit of copeptin, h-FABP, and cTnI was 0.40 pg/ml, 0.32 pg/ml, and 0.50 pg/ml respectively, which is at least one order of magnitude lower than most of the reported immunoassays (Yang et al., 2019).

Furthermore, they reported a temporal-spatial-color multi-resolved CL imaging method for multiplex immunoassay using a smartphone coupled with a microfluidic chip, which was performed by sequentially transporting coreactant H_2_O_2_ to the detection zones to initiate cobalt-based zeolitic imidazolate frameworks ZIF-67 catalyzed luminol-H_2_O_2_ CL and CL resonance energy transfer reactions (Li et al., 2020). The strategy was applied for multiplex analysis of three cancer biomarkers with a low LOD of pg/mL to fg/mL, good selectivity, and low-cost.

CLIA based on µPADs has become an attractive method because of its simplicity, high sensitivity, and high compatibility. However, sandwich-type CLIA need two antibodies and labeling technology, which suffers from multistep reactions and purifications, resulting in complex operation, high cost and long analytical time. Thus, It’s still challengeable to meet the requirement of rapid clinical diagnosis.

### 3.3 SERS-based microfluidic immunoassay

**FIGURE 6:**
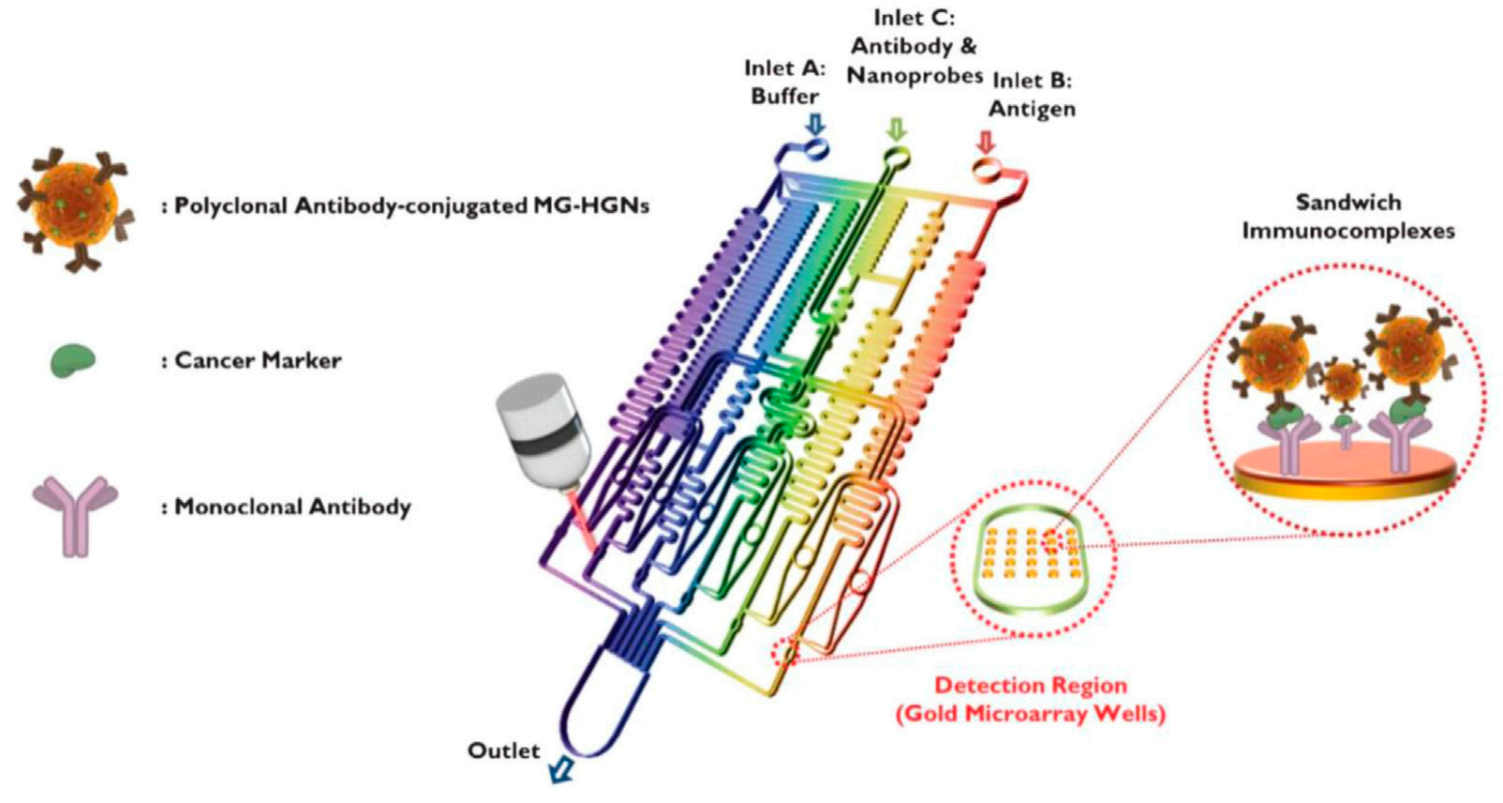
Schematic diagram of a gold array-embedded gradient chip for the SERS-based immunoassay. The illustrations in the enlarged circles represent the formation of sandwich immunocomplexes on the surface of 5*5 round gold wells embedded in the gradient channel (Lee et al., 2012).

SERS detection is a powerful spectroscopy technology by providing ultrasensitive and intrinsic chemical fingerprint information (Liu et al., 2018; Zuo et al., 2018). SERS-based assays are promising methods for immunoassays due to their rapid and sensitive analytical capabilities. SERS has inherent advantages over fluorescence, including minimal photobleaching effect, wide excitation wavelength range, and multiplexing capability (Gao et al., 2016). To achieve rapid immunoassay and reproducible SERS signals, SERS-based microfluidic chips have been developed to improve the performance of immunoassay, which involve of continuous microfluidic system and microdroplet microfluidics. The most common serial microfluidic immunoassays are performed in microfluidic channels ranging in size from 10 to 1,000 μm. Microfluidic channels can be analyzed in parallel with multiple samples simultaneously. Combined with SERS technology, microfluidic immunoassays can improve the performance of immunoassays in terms of sensitivity, response time, throughput, and overall cost (Wang et al., 2017). In continuous microfluidic system, the samples and reagents continuously flow in the microchannel. Choo’s group designed a SERS-based microfluidic immunoassay platform by combining a gradient microfluidic device with gold-patterned microarray wells (Figure 6) (Lee et al., 2012). A 5*5 gold microarray was embedded in the micro-channel to form sandwich immunocomplexes for SERS detection. They also utilized magnetic beads to form magnetic immunocomplexes which were trapped by yoke-type solenoids embedded within the microfluidic device. The SERS-based competitive immunoassay was performed within a microfluidic environment and the SERS signals of anthrax biomarker poly-γ-D-glutamic acid (PGA) were detected with a LOD of 100 pg/ml (Gao et al., 2015). Automating the immunoassay process in microfluidic channels increases the efficiency and control of manual operations. Recently, Li et al. reported a “magnetic focused” microfluidic immunoassay in which nanoprobes are magnetically focused to specific points in the microfluidic channel, enabling the enrichment of “hot spots” for SERS detection targeting carcinoembryonic antigen with a detection limit of 0.1 pM, achieving high sensitivity (Li et al., 2015). In addition, Kaminska et al. developed a gold-silver coated gallium nitride substrate as a novel immunofixation platform, which improved the efficiency of SERS-based microfluidic immunoassays and achieved a low detection limit of 0.01 IU/ml for hepatitis B virus antigen (Kaminska et al., 2015).

To reduce the sensor size and improve the portability of microfluidic sensor, Gao et al. proposed a novel pump-free microfluidic system for SERS-based immunoassay which applied a capillary pump to replace heavy syringe pump in conventional microfluidic system. Using this chip for rapid analysis of PSA biomarker, the LOD is estimated to be below 0.01 ng ml^−1^, with a good linear response in the range from 0.01 to 100 ng ml^−1^ (Gao et al., 2019). Furthermore, they developed another pump-free SERS-microfluidic chip for the simultaneous detection of creatine kinase MB isoenzyme (CK-MB) and cardiac troponin (cTnI) cardiac markers. In this study, the patterned SERS paper substrate was fabricated and then in-situ synthesized AuNPs on paper microchannel. The 3D fibre structure provided the capillarity force and compacted AuNPs deposition scaffold for sample flow and highly sensitive SERS detection, which simultaneously detected the concentrations of two cardiac markers ranging from 0.01 to 50 ng/ml (Gao et al., 2022).

**FIGURE 7:**
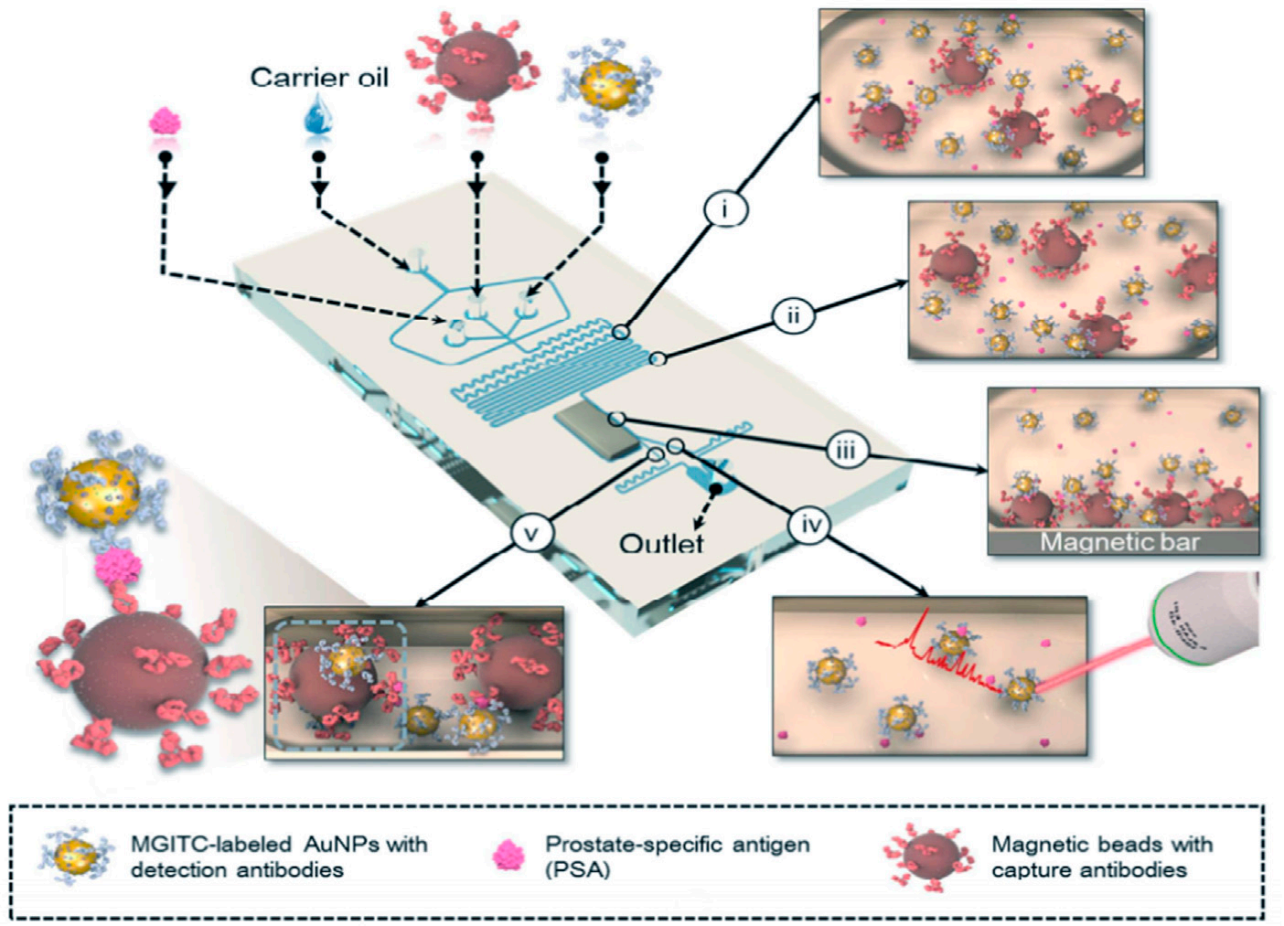
Schematic illustration of the SERS-based microdroplet sensor for wash-free magnetic immunoassay (Gao et al., 2016).

Unlike the continuous microfluidic systems, the microdroplet microfluidics focuses on creating discrete microdroplet by utilizing immiscible phases. Droplet microfluidic systems perform miniaturization reactions by separating reaction components into discrete microliters to picoliter volumes (Berry et al., 2019). It enables fast and high-throughput analysis without increasing the complexity and size of microfluidic chips. The advantages offered by this platform include reduced reagent consumption, precise control of reaction time, and the ability to synthesize highly homogeneous micro/nanostructures. Droplet microfluidics can be combined with SERS technology for fast and reproducible analysis. To date, the droplet SERS platform has been used in a variety of biochemical applications (Wang et al., 2017). Gao et al. reported a novel wash-free SERS-based microdroplet sensor for sensitive immunoassay of PSA biomarker (Figure 7). In this strategy, a magnetic bar was embedded in the microfluidic system to split droplets into two parts: one magnetic immunocomplexes and the other the free SERS tags. The presence of PSA target lead more SERS tags to immunocomplex in one droplet so that the SERS signals were able to be measured for quantitative evaluation of PSA marker. The SERS signals were measured at 174 droplets/minute and the LOD was estimated to be below 0.1 ng/ml (Gao et al., 2016).

**FIGURE 8:**
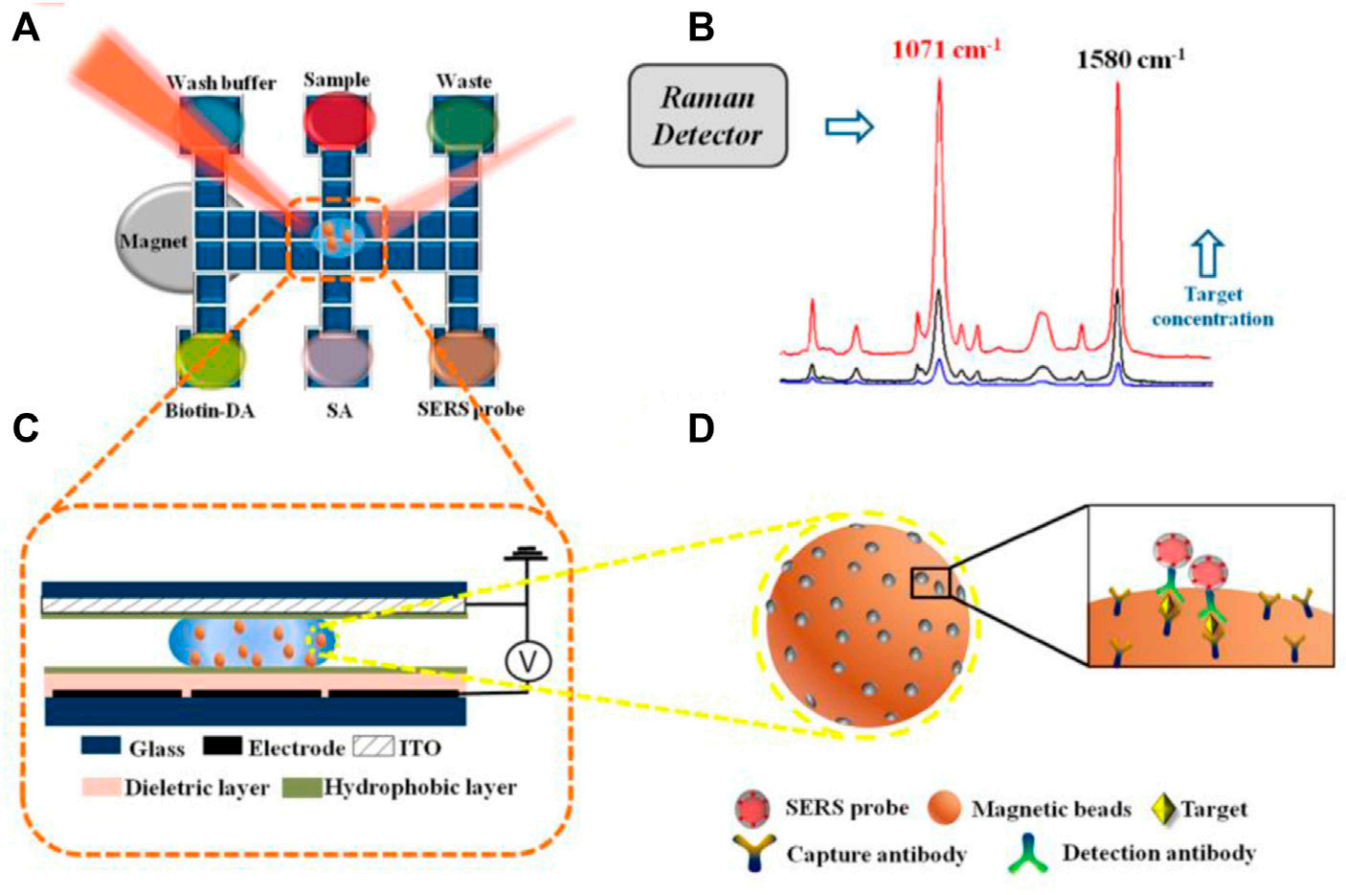
Schematic diagram of SERS immune analysis by DMF. **(A)** Illustration of DMF-SERS method and bottom plate of DMF chip. **(B)** Two characteristic Raman peaks of 4-MBA at 1071 cm^-1^ and 1580 cm^-1^. **(C)** Side view of DMF chip containing a droplet with magnetic beads. **(D)** Immunocomplex functionalized with SERS tags on magnetic beads.

The emerging of novel techniques promotes the development of microfluidic immunoassay chips. Wang et al. proposed an immunoassay method based on a novel digital microfluidics (DMF) and SERS for detection of disease biomarkers (Figure 8). Based on the principle of electrowetting on dielectric, droplets were individually driven to transport, merge, split, and dispense from reservoirs by applying electrical potentials to an array of electrodes, which greatly simplified the analysis process while reducing the risk of exposure to hazardous samples. Compared with the standard ELISA method, DMF-SERS method has high sensitivity (LOD 74 pg/ml) and good selectivity (Wang et al., 2018).

SERS based microfluidic chips provides an ideal mechanism for achieving homogeneous mixing, spatially defined detection areas, sensitive and reproducible measurements with low sample consumption. Because of the larger size of Raman detector, the miniaturization of SERS based microfluidic device is a challenge to achieve POCT immunoassay.

### 3.4 Electrochemical microfluidic immunoassay

**FIGURE 9:**
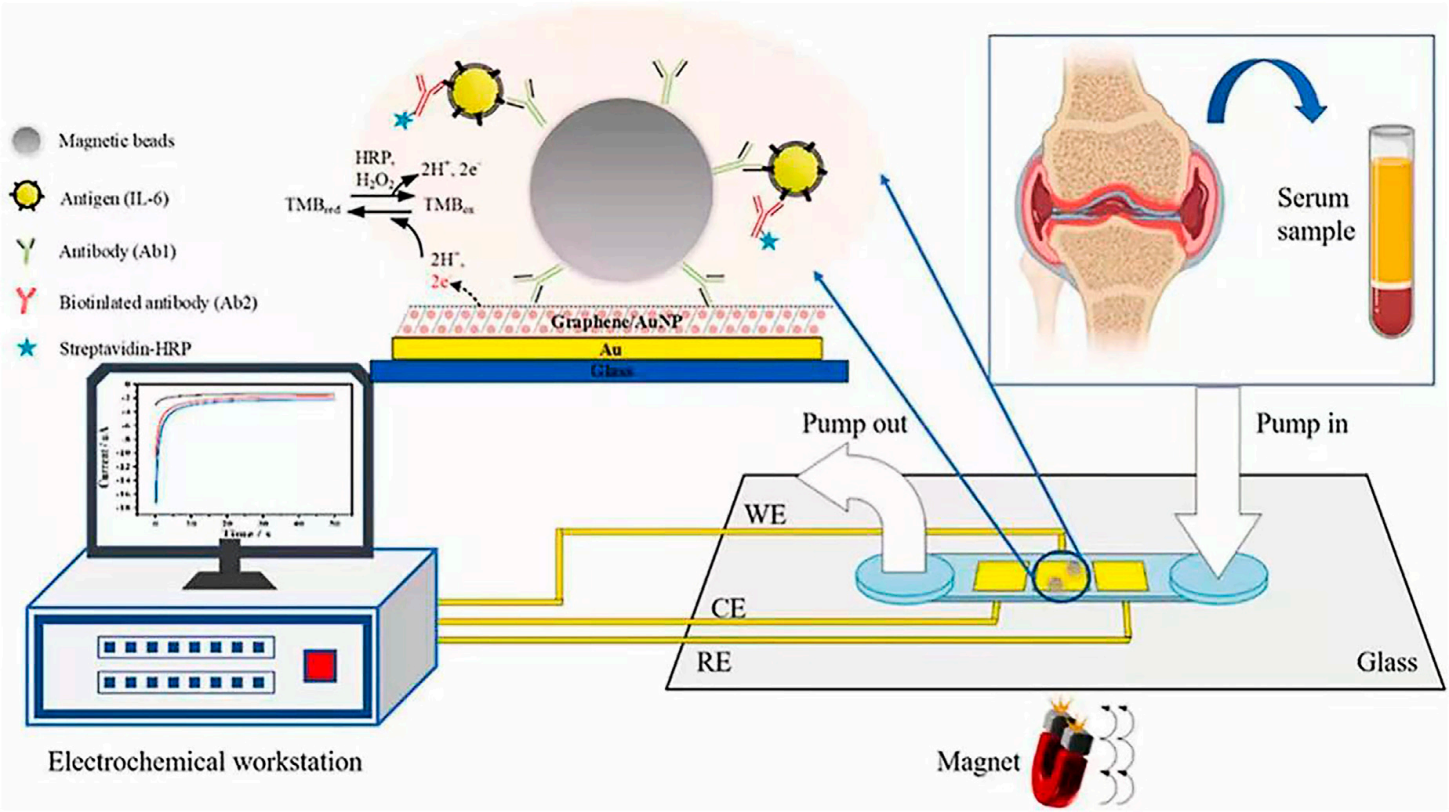
Microfluidic electrochemical magnetoimmunosensor based on hybrid of AuNPs and graphene (Zhang C. et al., 2022).

Electrochemical detection integrated with microfluidic devices are promising for immunoassay due to the benefits such as low sample consumption, low detection limit, and rapid, simple and portable assay (He et al., 2018a; Yang G. et al., 2018; Lai et al., 2019; Xie et al., 2020; He et al., 2021; Schmidt-Speicher and Länge, 2021; Wang et al., 2022). To improve the sensitivity of electrochemical microfluidic immunoassay, surface modification of electrodes in the microchannels are widely used in order to increase the surface area and binding ability (He et al., 2015; He et al., 2018b; Ning et al., 2018; Deng et al., 2021a; Deng et al., 2021b; Zhang et al., 2021). MatÃas et al. produced a gold nanoporous structure on the gold electrode in the microchannel by using dynamic hydrogen bubble template (DHBT) method, which obtained outstanding properties, like high specific surface area, large pore volume, good conductivity, and excellent electrochemical activity. The selectivity and sensitivity of the sensor were improved, achieving a LOD of 30 pg ml^−1^ and a variation coefficient less than 4.75% for the detection of SOX-2 cancer biomarker (Regiart et al., 2020). Jofre et al. reported an electrochemical microfluidic immunosensor by modifying the gold electrode with an ordered mesoporous carbon in chitosan to increase the sensitivity of detection (Jofre et al., 2020). Zhang et al. proposed a miniaturized electrochemical microfluidic immunosensor for interleukin-6 (IL-6) detection. The electrode was modified with gold nanoparticles and graphene to increase surface area, and magnetic beads were employed to concentrate immunoassay on the electrode (Figure 9). This magneto-immunosensor achieved a LOD of 0.42 pg/ml and a linear range from 0.97–250 pg/ml (Zhang C. et al., 2022).

**FIGURE 10:**
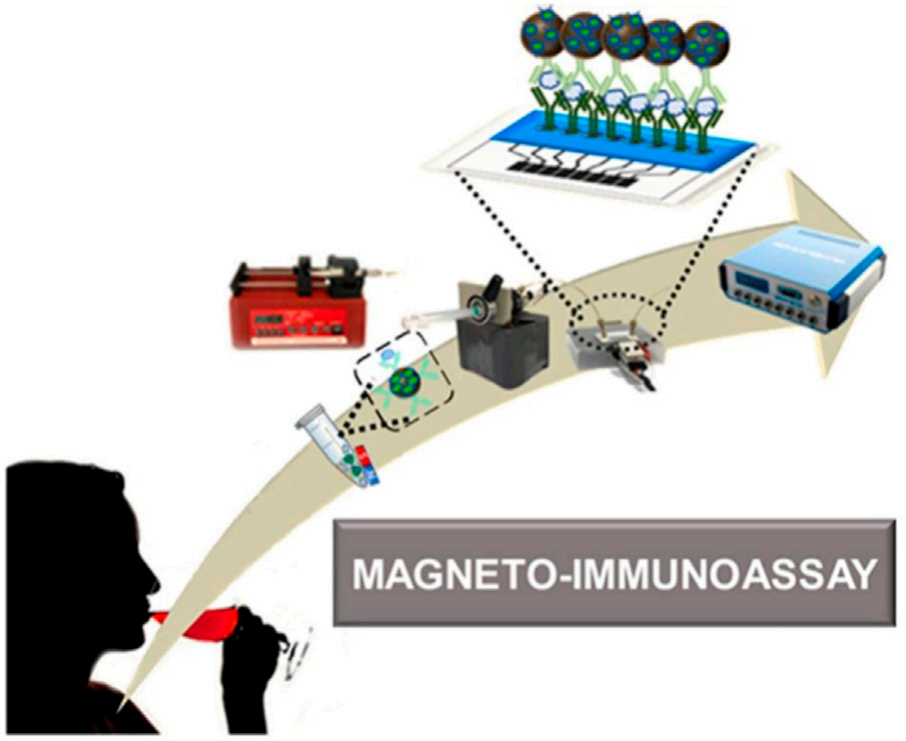
Schematic illustration of DEμD for OVA detection in wine samples (Baldo et al., 2021).

The advantages of electrochemical microfluidic devices such as being small and portable make them becoming the powerful candidates to construct POCT platform. In these devices, graphene oxide (GO) is usually coated on the electrodes using a screen-printing technique, which is beneficial to the covalent immobilization of proteins. Baldo et al. developed a disposable electrochemical microfluidic device (DEμD) for ultrasensitive detection of ovalbumin (OVA) in wine (Figure 10). In the DEμD, 8 GO-based working electrodes (8-WEs) integrated with horseradish peroxidase (HRP) and anti-OVA antibody modified magnetic beads to achieve sandwich assay. The magneto-immunoassay obtained a ultralow LOD of 0.2 fg ml^−1^ and a wide linear range of 0.01∼10 pg ml^−1^ for OVA detection, which allows eight simultaneous detections per device (Baldo et al., 2021).

**FIGURE 11:**
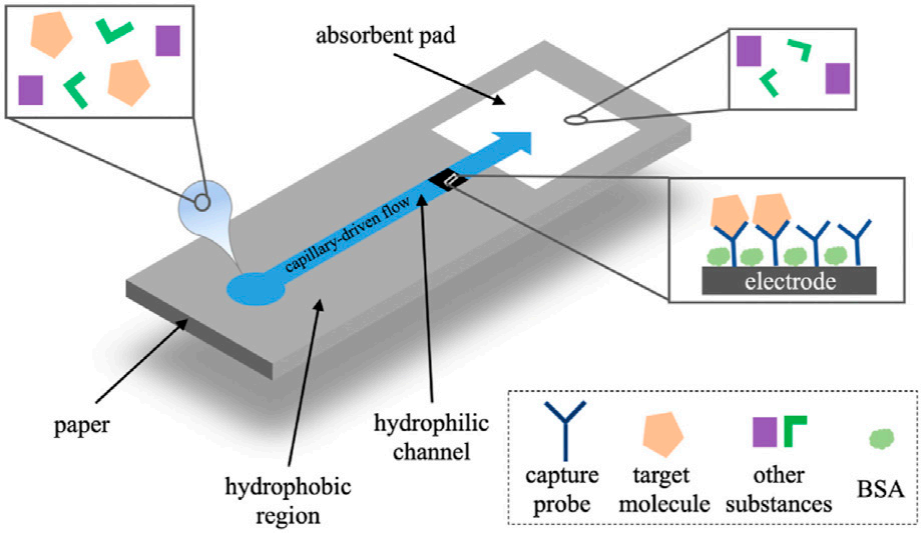
The operational principle of electrochemical μPADs (Yuan et al., 2022).

Paper-based analytical devices have the advantages of low cost, portable, eco-friendly, and high-throughput manufacturing, which makes it become an ideal alternative for POCT (Lim et al., 2019; Ruecha et al., 2019; Gutierrez-Capitan et al., 2020). Nowadays, microfluidic paper-based analytical devices (μPADs) integrated with electrochemical detection have been developed as typical POCT devices for immunoassay (Cate et al., 2015; Yuan et al., 2022). In electrochemical μPADs (Figure 11), samples flow through the micro-channel and the working electrode driven by the capillary. The target molecules are captured by the probes on the electrode and the electrochemical signals are detected (Yuan et al., 2022).

**FIGURE 12:**
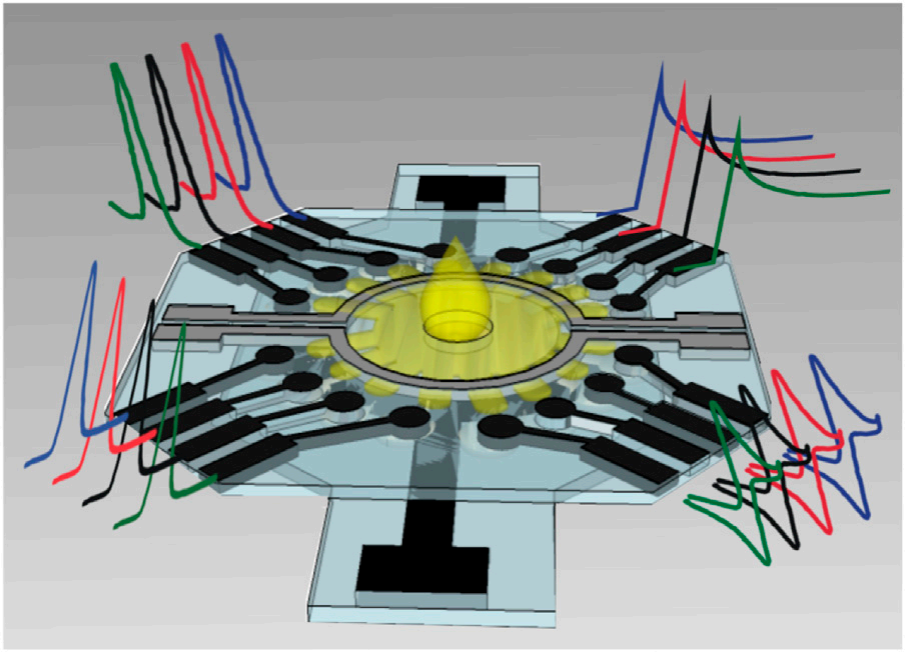
Schematic representation of multiplex μPAD based on 16 electrodes (Fava et al., 2019).

Cao et al. structured electrochemical μPADs for the detection of Human Chorionic Gonadotropin (HCG) based on the standard sandwich immunoreaction (Cao et al., 2017). Ruecha et al. developed a label-free electrochemical μPAD immunosensor in which the screen-printed electrodes were modified with polyaniline/graphene nanocomposites to provide high conductivity and large surface area, which obtained a LOD of 3.4 pg ml^−1^ and a linear range of 5–1,000 pg ml^−1^ for detection of human IFN-γ (Ruecha et al., 2019). For multiplex analysis, Wang et al. developed a multi-parameter μPAD for simultaneous detection of carcinoembryonic antigen (CEA) and neuron-specific enolase (NSE) (Yang et al., 2019). Fava et al. proposed a high-throughout μPAD based on 16 independent microfluidic channels for glucose determination in urine (Figure 12), obtaining a LOD of 3 × 10^−5^ mol L^−1^ and a linear range from 1.0 × 10^–4^ mol L^−1^ to 4 × 10^−2^ mol L^−1^ (Fava et al., 2019).

Electrochemical microfluidic devices provide an attractive strategy with low reagent consumption, sensitivity, ease of handling, cost-effectiveness, rapid analysis and miniaturization ability. Based on the simplicity of electrochemical detection, electrochemical microfluidic immunoasssay are promising in the development of POCT and multiplex detection devices.

In summary, different platforms have their own characteristics. Here, we list the representative configurations of each platform, including its detection mechanism, instrumentation, application, and detection limits, in Table 1 for a clear comparison.

**TABLE 1:** Different immunoassay applications based on microfluidic chips.

Detection principle	Instrumentation for integration	Applications	Results (LOD: Limit of detection; DR: Dynamic range)	References
Fluorescence	Fluorescence microscope	Enzymatic assay (glucose), magnetic bead-based immunoassay (lgE)	DR: 26–150 mg L^−1^ (glucose); LOD: 150 nM (lgE)	Vergauwe et al. (2011)
Fluorescence	Microplate reader	Rhombus-like Zn(OH)F array-based immunoassay (Cy3)	DR: 10 fM-100 pM; LOD: 9.3 fM	Zhao et al. (2021)
Fluorescence	Fluorescence microscope	ZnO nanowires-based immunoassay	LOD: 1 pg mL^−1^ (AFP) 100 fg mL^−1^ (CEA)	Guo et al. (2018)
Chemiluminescence	PMT	Magnetic bead-based immunoassay to detect insulin and interleukin-6	DR:8–1,000 pmol L^−1^ (insulin); 1.5–750 pg mL^−1^ (IL-6)	Sista et al. (2008)
Chemiluminescence	Photodiode	Magnetic bead-based immunoassay to separate and detect protein, bacteria and virus	LOD: 30 ng mL^−1^ (HSA); 4 × 10^4^ cfu mL^−1^ (*Bacillus atrophaeus*); 10^6^ cfu mL^−1^ (MS2 bacteriophage); 2 × 10^7^ cfu mL^−1^ (*Escherichia coli*)	Coudron et al. (2019)
Chemiluminescence	PMT	Magnetic bead-based immunoassay to quantitatively detect rubella IgG	DR: 0.15–100 μU mL^−1^ LOD: 0.15 μU mL^−1^	Fobel et al. (2014)
Chemiluminescence	PMT	Magnetic bead-based immunoassay to detect rubella virus (RV) IgG directly from blood	LOD: 1.9 IU mL^−1^	Dixon et al. (2020)
Surface enhanced Raman scattering (SERS)	Raman detector	Immunoassay on a DMF platform to detect avian influenza virus H5N1	LOD: 74 pg mL^−1^	Wang et al. (2018)
Electrochemistry	Electrochemical potentiostat	Magnetic bead-based immunoassay to detect interleukin-6	DR: 0.97–250 pg mL^−1^ LOD: 0.42 pg mL^−1^	Zhang et al. (2022a)
Electrochemistry	Electrochemical analyzer	Paper-based microfluidic device coupled with label-free electrochemical impedance immunosensor to detect human interferon-gamma (IFN-γ)	DR: 5–1,000 pg mL^−1^ LOD: 3.4 pg mL^−1^	Ruecha et al. (2019)

## 4 Conclusion

In recent years, microfluidic chip technology has been applied across many fields and disciplines, and biomedical applications are one of the focuses of current researches. The advantages brought by the unique physical and chemical properties of reactions in microchannels continue to promote the development of microfluidic chip technology. In this review, microfluidic chip technology is summarized from the aspects of chip materials, preparation technologies and applications in immunoassay. The emergence of new materials has improved some of the performance of microfluidic chip, but there are also some shortcomings which can not completely replace the earlier materials. In the future, the material development of microfluidic chips should fully consider the precision of processing technology, the processing cost and the ease of mass production. With the advantage of good compatibility with other detection techniques, the development of microfluidic chip should focus on integrated, automated, miniaturized and high throughput equipment. One obvious developing trend is portable microfluidic devices, which is great potential in POCT detection of biological samples with low consumption, real-time monitoring, simple operation and low cost. In addition, the designs of simultaneous detection of multiple biomarkers will be popular in the application of microfluidic chip as biosensor. With the rapid development of microfluidic devices, single performance microfluidic chip could not meet the current demand. The application of diverse and multifunctional manufacturing technologies is urgent, which requires not only the innovation of microfluidic chip materials, but also the integration of various technologies such as microfluidics and microelectronics. The multidisciplinary nature of microfluidic technology requires continuous and coordinated development between different fields. In addition, the actual sample analysis by microfluidic chip, such as sample input and pretreatment, still face the challenge. With the progress of various technologies and further research, microfluidic chips are of great potentials in commercial applications in the field of biological detection.
